# Fatty Acid Binding Proteins 3 and 4 Predict Both All-Cause and Cardiovascular Mortality in Subjects with Chronic Heart Failure and Type 2 Diabetes Mellitus

**DOI:** 10.3390/antiox12030645

**Published:** 2023-03-04

**Authors:** Ricardo Rodríguez-Calvo, Minerva Granado-Casas, Alejandra Pérez-Montes de Oca, María Teresa Julian, Mar Domingo, Pau Codina, Evelyn Santiago-Vacas, Germán Cediel, Josep Julve, Joana Rossell, Lluís Masana, Didac Mauricio, Josep Lupón, Antoni Bayes-Genis, Núria Alonso

**Affiliations:** 1 Vascular Medicine and Metabolism Unit, “Sant Joan” University Hospital, Institut de Investigació Sanitaria Pere Virgili (IISPV), 43204 Reus, Spain; 2 Research Unit on Lipids and Atherosclerosis, Universitat Rovira i Virgili, Institut de Investigació Sanitaria Pere Virgili (IISPV), 43204 Reus, Spain; 3 CIBER de Diabetes y Enfermedades Metabólicas Asociadas, Instituto de Salud Carlos III, 28029 Madrid, Spain; 4 Department of Nursing and Physiotherapy, Health Sciences Faculty, University of Lleida, IRBLleida, 25198 Lleida, Spain; 5 DAP-Cat Group, Unitat de Suport a la Recerca Barcelona, Institut Universitari d’Investigació en Atenció Primària Jordi Gol (IDIAP Jordi Gol), 08041 Barcelona, Spain; 6 Department of Endocrinology & Nutrition, Hospital Universitari Germans Trias i Pujol, 08916 Badalona, Spain; 7 Heart Failure Clinic and Cardiology Service, University Hospital Germans Trias i Pujol, 08916 Badalona, Spain; 8 Institut de Recerca de l’Hospital de la Santa Creu i Sant Pau, 08041 Barcelona, Spain; 9 Institut d’Investigació Biomèdica de l’Hospital de la Santa Creu i Sant Pau, IIB Sant Pau, 08041 Barcelona, Spain; 10 Department of Endocrinology & Nutrition, Hospital de la Santa Creu i Sant Pau, IIB-Sant Pau, 08041 Barcelona, Spain; 11 Faculty of Medicine, University of Vic & Central University of Catalonia, 08500 Vic, Spain; 12 Department of Medicine, Universitat Autonoma de Barcelona, 08023 Barcelona, Spain; 13 CIBERCV, Instituto de Salud Carlos III, 28029 Madrid, Spain

**Keywords:** FABP3, FABP4, chronic heart failure, diabetic patients, all-cause death, cardiovascular death, rehospitalization

## Abstract

Subjects with type 2 diabetes mellitus (T2D) are at increased risk for heart failure (HF). The cardiac-specific (FABP3) and adipose-tissue-specific (FABP4) types of the fatty acid binding proteins have been associated with both all-cause and cardiovascular (CV) mortality. The aim of this study was to explore the prognosis value of FABP3 and FABP4 in ambulatory subjects with chronic HF (CHF), with and without T2D. A prospective study involving 240 ambulatory CHF subjects was performed. Patients were followed-up for a mean of 5.78 ± 3.30 years and cause of death (if any) was recorded. Primary endpoints were defined as all-cause and CV death, and a composite endpoint that included CV death or hospitalization for HF was included as a secondary endpoint. Baseline serum samples were obtained and the serum FABP3 and FABP4 concentrations were assessed by sandwich enzyme-linked immunosorbent assay. Survival analysis was performed with multivariable Cox regressions, using Fine and Gray competing risks models when needed, to explore the prognostic value of FABP3 and FABP4 concentrations, adjusting for potential confounders. Type 2 diabetes mellitus was highly prevalent, accounting for 47.5% for total subjects with CHF. Subjects with T2D showed higher mortality rates (T2D: 69.30%; non-T2D: 50.79%, *p* = 0.004) and higher serum FABP3 (1829.3 (1104.9–3440.5) pg/mL vs. 1396.05 (820.3–2362.16) pg/mL, *p* = 0.007) and FABP4 (45.5 (27.6–79.8) ng/mL vs. 34.1 (24.09–55.3) ng/mL, *p* = 0.006) concentrations compared with non-T2D CHF subjects. In the whole study cohort, FABP3 was independently associated with all-cause death, and both FABP3 and FABP4 concentrations were associated with CV mortality. The predictive values of these two molecules for all-cause (FABP3: HR 1.25, 95% CI 1.09–1.44; *p* = 0.002. FABP4: HR 2.21, 95% CI 1.12–4.36; *p* = 0.023) and CV mortality (FABP3: HR 1.28, 95% CI 1.09–1.50; *p* = 0.002. FABP4: HR 4.19, 95% CI 2.21–7.95; *p* < 0.001) were only statistically significant in the subgroup of subjects with T2D. Notably, FABP4 (HR 2.07, 95% CI 1.11–3.87; *p* = 0.022), but not FABP3, also predicted the occurrence of the composite endpoint (death or hospitalization for HF) only in subjects with T2D. All these associations were not found in CHF subjects without T2D. Our findings support the usefulness of serum FABP3 and FABP4 concentrations as independent predictors for the occurrence of all-cause and CV mortality in ambulatory subjects with CHF with T2D.

## 1. Introduction

Increasing evidence has shown a greater risk for heart failure (HF) associated with the presence of type 2 diabetes mellitus (T2D) [1]. Indeed, HF is one of the main cardio-vascular (CV) manifestations reported in subjects with T2D [2]. Despite this, the prognosis of subjects with HF and T2D is elusive [3]. In this regard, the enhanced mortality risk in these subjects cannot be fully explained by established risk factors [4,5,6,7]. Therefore, stratification of the mortality risk related to HF remains a challenge for these subjects and additional HF biomarkers among subjects with T2D should be considered.

Metabolic disturbances, including impaired glucose and fatty acid metabolism, have been related to increased risk for HF independently of coronary artery disease [8]. Enhanced oxidative stress, mitochondrial dysfunction and cardiomyocyte apoptosis are among the main molecular mechanisms underlying myocardial dysfunction [9]. Accumulating evidence suggests a role for serum circulating molecules that may behave as sensors of metabolic alterations and might directly contribute to increased risk of HF in subjects with T2D. In this context, several members of the Fatty Acid Binding Protein (FABP) family have been linked to metabolic diseases related to cardiac disorders [10]. Members of this family are intracellular lipid transporters that take part in the intracellular regulation of lipid trafficking and their responses. Specifically, the cardiac-specific fatty acid binding protein (FABP3) has been related to the control of cardiac insulin resistance [11] and fatty acid uptake [12]. Another form of FABP, adipose-tissue-specific (FABP4), exhibits cardio-depressant effects [13] and participates in the trans-endothelial transport of nutrients to the cardiomyocyte [14], directly impacting insulin signaling in cardiac cells [15]. Both FABP3 and FABP4 have been described as circulating biomarkers of several cardiac and metabolic disturbances. FABP3 is rapidly released into the bloodstream after acute myocardial injury [16,17,18]. FABP3 elevations have also been related to different cardiac pathologies, including several cardiomyopathies, acute coronary syndrome (ACS) and HF [19] and proposed as a silent biomarker for the progression of myocardial damage in subjects with insulin resistance [20]. On the other hand, FABP4 has also been related to HF and CV disease [10,15,21,22,23,24,25,26,27,28]. Specifically, serum FABP4 concentrations correlate positively with the HF biomarker N-terminal fragment of pro-B-type natriuretic peptide (NT-proBNP), this association being even stronger in subjects with diabetes and HF [23]. Recently, FABP4 has also been related to ectopic fat accumulation in the heart [15], one of the main precursors of myocardial dysfunction due to diabetes [29,30,31,32,33].

Both FABP3 and FABP4 have been linked to oxidative stress. For instance, circulating FABP3 has been positively related to oxidative stress biomarkers, including malondialdehyde (MDA) and asymmetric dimethylarginine (ADMA), and inversely associated with the total antioxidant capacity (TAC) in patients with carbon-monoxide-induced cardiotoxicity [34]. On the other hand, experimental studies performed in FABP4-deficient mice showed a decline in oxidative stress during myocardial ischemia/reperfusion (MI/R) injury and diabetes-induced cardiac dysfunction, as revealed by concomitant activation of the endothelial nitric oxide synthase/nitric oxide (eNOS/NO) pathway and reduced superoxide anion production [35]. Therefore, both FABP3 and FABP4 may directly impact the disease progression through oxidative stress regulation.

An increasing body of evidence supports the notion that both FABP3 and FABP4 serum concentrations can predict both all-cause [36,37,38,39,40,41,42] and CV mortality [40,41,42,43,44,45,46,47,48,49,50,51,52,53,54,55,56,57,58]; however, the potential role of these FABPs as predictive biomarkers for the mortality risk among subjects with T2D and chronic HF (CHF) has not been explored yet. Thus, the aim of this study was to assess the prognostic value of these two FABPs (i.e., FABP3 and FABP4) for both all-cause and CV mortality in outpatient CHF subjects with T2D.

## 2. Materials and Methods

### 2.1. Study Population

The current research was performed in a subset of a well-characterized ambulatory cohort of subjects with CHF, prospectively admitted in a structured ambulatory multidisciplinary HF unit [59,60]. Specifically, samples from 240 outpatients with CHF referred to the HF unit were included in the study. Heart failure was diagnosed according to the European Society of Cardiology guidelines regardless of etiology. Baseline serum samples were obtained via centrifugation from venous blood samples and stored at −80 °C for further analysis, avoiding freeze–thaw cycles. Clinical echocardiograms were performed at baseline, and left ventricular dimensions and function were determined according to guidelines [61,62]. Patients were followed-up until death or end of follow-up (if alive), and causes of death (if any) were recorded. All-cause and CV death were defined as the primary endpoints of the study. A death was considered as CV when it was due to HF (worsening HF or treatment-resistant HF in the absence of another cause), sudden cardiac death (any unexpected death, witnessed or not, of a previously stable patient with no evidence of worsening HF or any other known cause of death), myocardial infarction, stroke, secondary to a CV procedure (post-diagnostic or post-therapeutic) or other CV causes (e.g., rupture of an aneurysm, peripheral ischemia or aortic dissection). Cardiovascular death and HF rehospitalization were further included as composite endpoint. Nine patients were lost during follow-up and appropriately censored.

All participants provided written informed consent. The study was approved by Local Ethics Committee of the Hospital Universitari Germans Trias i Pujol (code: EO 10-076) and was performed according to the ethical standards outlined in the Declaration of Helsinki [63].

### 2.2. Clinical and Biochemical Data

Anthropometric and clinical data were obtained at the point of study inclusion and were described elsewhere [60].

### 2.3. Serum FABPs Determination

Serum concentrations of FABP3 and FABP4 (Biovendor, Brno, Czech Republic) were determined in duplicate using commercial sandwich enzyme-linked immunosorbent assay kits (intra- and inter-assay coefficients of variation were estimated <5%).

### 2.4. T2D Diagnosis

A diagnosis of T2D was made when one of the following criteria were met: (1) a diagnosis of T2D was previously established and recorded in the patient’s electronic history, (2) fasting plasma glucose ≥ 126 mg/dL or HbA1c ≥ 6.5% identified by laboratory testing [19] or (3) the patient had a current prescription for oral hypoglycemic medication or insulin. All the included patients in this study had type 2 diabetes.

### 2.5. Statistical Analysis

The Kolmogorov–Smirnov test was used to determine the normality of the continuous variables. Continuous variables were expressed as median and interquartile range, unless otherwise indicated. Categorical variables are expressed as numbers with percentages. Differences between patients were analyzed by the Chi-squared test, Student’s *t* test and Mann–Whitney U test, as required.

The association of FABP3 and FABP4 with all-cause and CV mortality, or the composite endpoint (i.e., CV death and HF hospitalization), was evaluated using a multivariable Cox regression analysis approach. The primary endpoints were considered as the dependent variables and the selected relevant clinical variables (i.e., age, sex, diabetes, ischemic etiology, New York Association (NYHA) functional class, time of evolution, FEECO (ejection fraction on echocardiography) NT-ProBNP and obesity) plus FABP3 or FABP4 as independent covariables. Competing risks models using the Fine and Gray method were realized with CV mortality and the composite endpoint (i.e., CV mortality or HF hospitalization). FABP3 and FABP4 analyses were performed for each 1 ng/mL or 1 ng/dL increase, respectively. Statistical analyses were performed using STATA V.16.0 (College Station, TX, USA). Differences were considered statistically significant with a two-sided *p* < 0.05.

## 3. Results

The baseline characteristics of the study population and a comparison of the clinical and biochemical parameters of patients with CHF with and without T2D are shown in Table 1. Out of 240 subjects with CHF included, 170 were men and 70 were women. The median age of the study population was 69 (59–77) years. Approximately 14.7% of subjects were usual smokers and 43.3% ex-smokers. Type 2 diabetes was present in 114 (47.5%) of subjects. The percentages of subjects with hypertension and hypercholesterolemia were higher in patients with T2D compared with those without T2D (77.2% vs. 57.9%, *p*-value = 0.002; 80.7% vs. 49.2%, *p*-value < 0.001, respectively). Upon inclusion, 73.7% of subjects with CHF and T2D were receiving oral antidiabetic drugs and 62.3% were under insulin treatment. The percentage of subjects with NYHA functional classes III-IV was higher in subjects with T2D compared with those without T2D (29.0% vs. 15.9%, *p*-value < 0.015). Subjects with T2D showed lower serum concentrations of total-, HDL- and LDL-cholesterol and increased serum concentrations of creatinine and NT-proBNP, compared with subjects without T2D. Additionally, subjects with T2D also showed increased serum concentrations of FABP3 (1.3-fold, *p*-value = 0.007) and FABP4 (1.3-fold, *p*-value = 0.006) compared with subjects without T2D. No significant correlations were found between lipid parameters (i.e., total-, LDL- and HDL-cholesterol and triglycerides) with FABP3 and FABP4 in T2D patients (Supplementary Figure S1). Urate was determined as a surrogate biomarker for oxidative status [64,65,66]. Whereas both FABP3 and FABP4 were found positively correlated with urate in non-T2D individuals (FABP3: ρ = 0.221, *p*-value < 0.013; FABP4: ρ = 0.195, *p*-value < 0.029), non-significant correlations were found in T2D patients (FABP3: ρ = 0.022, *p*-value < 0.820; FABP4: ρ = −0.038, *p*-value < 0.692) (Supplementary Figure S2). The mortality rate was higher in subjects with T2D compared with those without T2D (18.5%, *p*-value = 0.004 vs. non-T2D). During a mean follow-up period of 5.78 ± 3.30 years, 143 patients died. The average years of follow-up until death (5.22 (2.02–8.17) vs. 7.13 (3.27–8.90), *p*-value = 0.009) or the composite endpoint (death or readmission) (2.57 (0.61–6.81) vs. 5.72 (0.90–8.31), *p*-value = 0.006) were lower in T2D than in non-T2D subjects.

**Figure 1:**
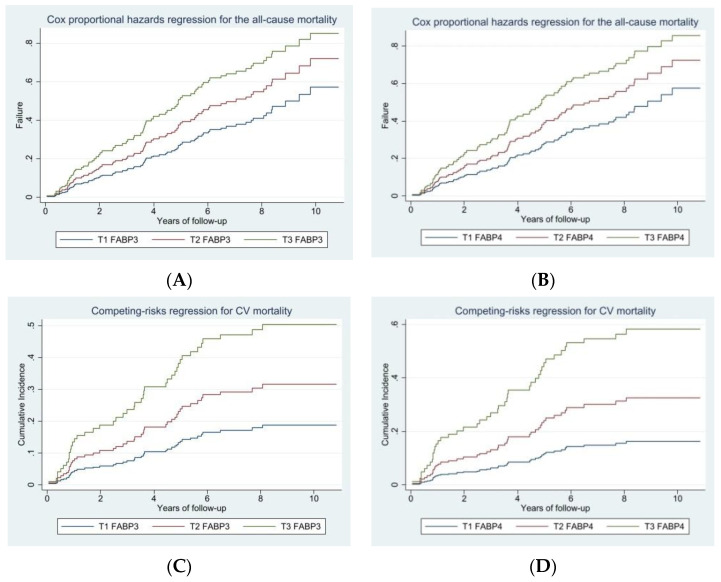
Both all-cause (**A**,**B**) and CV (**C**,**D**) mortality stratified by the tertiles of serum FABP3 (**A**,**C**) and FABP4 (**B**,**D**) in T2D patients. Data adjusted by age, gender, ischemic etiology, NYHA III and IV, HF duration, LVEF, NTproBNP, obesity and eGFR were expressed as incidence of mortality by Cox proportional hazards and competing-risks regression. Overall *p*-value < 0.05.

The rate of all-cause mortality increased along with FABP3 (Figure 1A) and FABP4 (Figure 1B) serum tertiles among subjects with T2D. Similarly, the rate of CV death was also increased with FABP3 (Figure 1C) and FABP4 (Figure 1D) tertiles.

**Figure 2:**
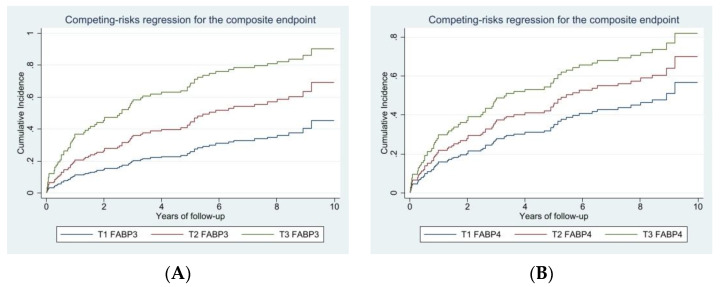
Composite endpoint (death or rehospitalization) stratified by the tertiles of serum FABP3 (**A**) and FABP4 (**B**) in T2D patients. Data adjusted by age, gender, ischemic etiology, NYHA III and IV, HF duration, LVEF, NTproBNP, obesity and eGFR are expressed as incidence of death or rehospitalization by competing-risks regression. Overall *p*-value < 0.01.

In line with these observations, in subjects with T2D, multivariable Cox models revealed both FABP3 and FABP4 as independent predictors for the occurrence of all-cause mortality (FABP3: HR 1.25, 95% CI 1.09–1.44, *p*-value = 0.002; FABP4: HR 2.21, 95% CI 1.12–4.36, *p*-value = 0.023, respectively) (Table 2) and CV death (FABP3: HR 1.28, 95% CI 1.09–1.50, *p* = 0.002; FABP4: HR 4.19, 95% CI 2.21–7.95, *p*-value < 0.001, respectively) (Table 3). Nevertheless, in subjects without T2D, serum FABP3 and FABP4 concentrations were unable to predict both all-cause (Supplementary Table S1) and CV (Supplementary Table S2) mortality. Additionally, FABP4 (HR 2.07, 95% CI 1.11–3.87; *p*-value = 0.022), but not FABP3, predicted the occurrence of the composite endpoint (CV death or rehospitalization for HF) in subjects with CHF and T2D (Table 4), but not in subjects without T2D (Supplementary Table S3). Indeed, the composite endpoint rate also increased along with serum FABP3 (Figure 2A) and FABP4 (Figure 2B) tertiles in subjects with T2D. Finally, when both subjects with and without T2D were included in the analysis, the occurrences of all-cause (Supplementary Table S4) death were predicted by FABP3 and the occurrence of CV mortality (Supplementary Table S5) was predicted by both FABP3 and FABP4. Additionally, none of the studied FABPs were able to predict the occurrence of the composite endpoint in the whole cohort (Supplementary Table S6).

Multivariable models were adjusted for clinically relevant variables. AUC for FABP3 = 0.8644. AUC for FABP4 = 0.8657. FABP3: fatty acid binding protein 3; FABP4: fatty acid binding protein 4; HF: heart failure; NYHA: New York heart association; LVEF: left ventricular ejection fraction; NTproBNP: pro-B-type natriuretic peptide; eGFR estimated glomerular filtration rate (CKD-EPI equation).

Multivariable models were adjusted for clinically relevant variables. AUC for FABP3 model = 0.7532. AUC for FABP4 model = 0.7428. FABP3: fatty acid binding protein 3; FABP4: fatty acid binding protein 4; HF: heart failure; NYHA: New York heart association; LVEF: left ventricular ejection fraction; NTproBNP: pro-B-type natriuretic peptide; eGFR estimated glomerular filtration rate (CKD-EPI equation); SHR: Subdistribution Hazard Ratio.

Multivariable models were adjusted for clinically relevant variables. AUC for FABP3 model = 0.7131. AUC for FABP4 model = 0.7188. FABP3: fatty acid binding protein 3; FABP4: fatty acid binding protein 4; HF: heart failure; NYHA: New York heart association; LVEF: left ventricular ejection fraction; NTproBNP: pro-B-type natriuretic peptide; eGFR estimated glomerular filtration rate (CKD-EPI equation); SHR: Subdistribution Hazard Ratio.

## 4. Discussion

Subjects with CHF and T2D frequently display a poor prognosis [3]. In these subjects, the risk stratification of mortality is a challenging goal as it cannot be fully predicted by established risk factors [4,5,6,7]. Both FABP3 and FABP4 have been directly linked to a wide range of metabolic and cardiac disturbances, including HF [10,15,19,21,22,23,24,25,26,27,28]. Remarkably, increased serum concentrations of these molecules have been associated with myocardial alterations in subjects with impaired insulin signaling [15,20]. On the other hand, accumulating experimental evidence shows that both molecules can actively promote cardiac remodeling, leading to myocardial dysfunction [11,15].

The role of FABP3 and FABP4 as independent predictors of mortality has been reported in subjects with pulmonary embolism [46,47] and after acute coronary syndrome (ACS) [36,37,38], and all-cause death increased together with increasing FABP3 tertiles in subjects with stable angina [39]. Noteworthily, increased circulating FABP4 concentrations were found significantly associated with all-cause death in subjects with T2D [40,41], and all-cause mortality was associated with the highest tertile of FABP4 concentrations in subjects with peripheral arterial disease [42].

In the present study, a comprehensive Cox regression model was built in order to further analyze the potential role of both FABP3 and FABP4 as all-cause mortality predictors in a cohort of ambulatory patients with CHF. Serum concentrations of both molecules were higher in patients with CHF and T2D compared with patients with CHF without T2D. In our models, FABP3, but not FABP4, was identified as an independent predictor of the all-cause death in the whole study cohort. Remarkably, when only CHF subjects with T2D were considered, both FABP3 and FABP4 predicted the occurrence of all-cause mortality but were unable to predict the all-cause mortality in the subgroup of subjects without T2D.

Focusing on CV mortality prediction, previous studies identified that low concentrations of FABP3 may predict CV death in combination with high BNP concentrations in patients with non-ischemic dilated cardiomyopathy [43]. Moreover, FABP3 has been defined as an independent predictor of CV events, including CV death, in subjects with suspected ACS [44], patients with HF and preserved ejection Fraction (HFpEF) [45] and in subjects with stable coronary artery disease and impaired glucose metabolism [48]. On the other hand, circulating FABP4 has been proposed as an independent predictor of CV mortality in the general population [57] and in patients with end-stage renal disease [52], peripheral arterial disease [42], coronary heart disease [52], stable angina undergoing percutaneous coronary intervention [54], ischemic stroke [49] and T2D [40,41,55]. In addition, circulating FABP4 concentrations have been reported to predict the risk of CV mortality among older adults with and without established CV disease [56] and associated with the risk of sudden cardiac death in older non-T2D individuals [53]. Moreover, FABP4 changes over time have been associated with adverse clinical outcomes, including CV death, in ambulatory patients with CHF [58]. In this context, we performed a competitive risk-regression model in order to explore the role of FABP3 and FABP4 predicting the occurrence of CV mortality. To our knowledge, this is the first report that FABP3 has strong predictive value for CV death in ambulatory CHF subjects with T2D. Noteworthily, it failed predicting CV death in subjects without T2D. Similarly, FABP4 predicted CV mortality in subjects with T2D, but not in non-T2D individuals. To further confirm this notion, additional studies were performed in order to explore the potential predictive value of FABP3 and FABP4 for a composite endpoint, including CV death and readmission, for HF. FABP4, but not FAPB3, was able to predict composite endpoint in the subset of CHF subjects with T2D, but not in subjects without T2D.

In the metabolic context of T2D, the energy substrates of cardiomyocytes to produce metabolic energy switch from glucose to fatty acid. The increased use of fatty acids for energy production in mitochondria is frequently associated with increased reactive oxygen species (ROS) production, which leads to enhanced oxidative stress in diabetic cardiomyocytes. The accumulation of ROS profoundly affects normal cardiomyocyte physiology and function, leading to reduced cardiac contractibility and maladaptive cardiac response [67]. In this context, both FABP3 and FABP4 may directly impact the disease through oxidative stress regulation. Actually, both FABPs directly contribute to the fatty acids transport and, thus, may further fuel mitochondria. The serum levels of FABP3 have been found to be directly correlated to some oxidative stress biomarkers, such as MDA and ADMA, and inversely correlated to total TAC in patients with carbon-monoxide-induced cardiotoxicity [34]. On the other hand, FABP4 has been identified as a key molecule in oxidative stress during MI/R injury and diabetes-induced cardiac dysfunction in FABP4-knockout mice [35]. Additionally, FABP4 deficiency also led to activation of the eNOS/NO pathway and reduction in superoxide anion production [35]. Noteworthily, we used the serum concentrations of urate as a surrogate biomarker of oxidative status [64,65,66]; however, neither FABP3 nor FABP4 were associated with urate in T2D patients. Nevertheless, further molecular analyses are warranted in order to fully characterize the contribution of FABP3 and FABP4 to cardiac disturbances related to oxidative stress.

Our study has some limitations. First, it was performed in a subset of the general population attending a single-center HF unit in a tertiary hospital, and it is not possible to rule out the possibility of bias due to selection. The relatively small sample size attenuated the impact of the results. Unfortunately, data on insulin, as well as oxidative parameters other than urate were unavailable in our cohort data sets. Additionally, the retrospective nature of our study precludes the extrapolation of causal relationships from our data. Nevertheless, our data are in line with increasing evidence suggesting a prognosis value of both FABP3 and FABP4 in a wide range of pathologies. Finally, although several adjusted multivariate models were performed, additional confounders may have had an impact on the results.

## 5. Conclusions

Overall, our findings strongly support the role of both serum FABP3 and FABP4 as independent predictors for the occurrence of all-cause and CV mortality in ambulatory subjects with T2D and CHF.

## Figures and Tables

**Table 1:** Clinical characteristics of the study subjects.

Characteristics	All N = 240	T2D N = 114	Non-T2D N = 126	* *p*-Value
Age, years	69.0 [58.5–77.0]	71.0 [63.0–77.0]	66.5 [53.0–78.0]	0.063
Sex, women	70 (29.17)	36 (31.58)	34 (26.98)	0.434
Ethnicity, Caucasian	234 (97.50)	111 (97.37)	123 (97.62)	0.388
Smoking				
Current smoker	34 (14.70)	14 (12.28)	20 (15.87)	0.425
Former smoker	104 (43.33)	48 (42.11)	56 (44.44)	0.715
BMI, Kg/m^2^	26.47 [23.59–30.21]	27.06 [23.95–31.24]	26.04 [23.31–28.84]	0.075
Hypertension	161 (67.08)	88 (77.19)	73 (57.94)	0.002
Hypercholesterolemia	154 (64.17)	92 (80.70)	62 (49.21)	<0.001
Oral antidiabetic drugs	84 (35.00)	84 (73.68)	0 (0)	<0.001
Insulin treatment	71 (29.58)	71 (62.28)	0 (0)	<0.001
Ischemic heart disease	100 (41.67)	54 (47.37)	46 (36.51)	0.088
NYHA III and IV	53 (22.08)	33 (28.95)	20 (15.87)	0.015
LVEF	34.00 [25.00–42.00]	34.00 [28.00–42.00]	34.00 [24.00–44.00]	0.824
HF duration, months	6.00 [2.00–45.00]	8.00 [2.00–39.00]	5.00 [2.00–48.00]	0.413
Admission for heart failure	105 (43.75)	57 (50.00)	48 (38.10)	0.063
Ischemic etiology	117 (48.75)	66 (57.89)	51 (40.48)	0.007
Total cholesterol, mg/dL	172.85 [141.23–210.88]	159.66 [133.86–198.66]	178.87 [149.77–221.40]	0.002
LDL cholesterol, mg/dL	89.36 [74.42–106.60]	87.74 [68.89–116.28]	104.72 [83.21–131.61]	<0.001
HDL cholesterol, mg/dL	46.94 [41.66–54.25]	43.46 [36.86–52.77]	48.69 [41.13–55.87]	0.005
Triglycerides, mg/dL	120.46 [85.91–170.50]	126.21 [4.14–173.60]	114.70 [86.79–162.97]	0.550
Creatinine, mg/dL	1.20 [1.00–1.71]	1.40 [1.04–2.00]	1.10 [0.96–1.60]	0.004
Urate, mg/dL	6.50 [6.50–6.50]	6.50 [6.50–6.50]	6.50 [6.50–6.50]	0.099
eGFR, mL/min/1.73 m^2^	54.20 [34.88–78.52]	48.59 [28.06–69.58]	67.19 [38.99–85.62]	0.001
NTproBNP, ng/L	2142.50 [763.50–5050.00]	2675.5 [1104.00–5780.00]	1820.50 [593.00–3885.00]	0.005
FABP3, pg/mL	1596.09 [971.60–2894.00]	1829.33 [1104.92–3440.49]	1396.05 [820.3–2362.16]	0.007
FABP4, ng/mL	39.90 [25.98–66.63]	45.5 [27.62–79.82]	34.1 [24.09–55.3]	0.006
Deaths	143 (59.58)	79 (69.30)	64 (50.79)	0.004
Death follow-up, years	6.67 [2.85–8.55]	5.22 [2.02–8.17]	7.13 [3.27–8.90]	0.009
CV mortality	73 (31.47)	44 (40.00)	29 (23.77)	0.008
CV mortality and/or admission for HF	134 (57.26)	77 (69.37)	57 (46.34)	0.002
CV mortality and/or admission for HF follow-up, years	3.76 [0.79–7.81]	2.57 [0.61–6.81]	5.72 [0.90–8.31]	0.006

Data are shown as n (%) for categorical variables and median [interquartile range] for continuous variables. * *p*-values between T2D and non-T2D are indicated. T2D: type 2 diabetes mellitus; BMI: body mass index; NYHA: New York heart association; FEECO: ejection fraction on echocardiography; LDL: low density lipoproteins; HDL; high density lipoproteins; eGFR estimated glomerular filtration rate (CKD-EPI equation); HF: heart failure; NTproBNP: pro-B-type natriuretic peptide; FABP3: fatty acid binding protein 3; FABP4: fatty acid binding protein 4; CV: cardiovascular; HF: heart failure.

**Table 2:** Cox regression models for the FABP3, FABP4 concentration and all-cause mortality in subjects with T2D.

	HR (95% CI)	*p*-Value	HR (95% CI)	*p*-Value
FABP3, ng/mL	1.25 (1.09–1.44)	0.002	-	-
FABP4, ng/dL	-	-	2.21 (1.12–4.36)	0.023
Age, years	1.04 (1.01–1.07)	0.003	1.04 (1.01–1.06)	0.004
Sex, women	0.74 (0.39–1.39)	0.345	0.61 (0.32–1.19)	0.149
Ischemic etiology	2.07 (1.20–3.57)	0.009	2.15 (1.22–3.80)	0.008
NYHA III and IV, %	1.71 (0.97–3.01)	0.062	1.83 (1.05–3.21)	0.034
HF duration, years	1.00 (1.00–1.01)	0.124	1.00 (1.00–1.01)	0.149
LVEF	1.02 (0.99–1.04)	0.198	1.01 (0.99–1.04)	0.255
NTproBNP, ng/L	1.00 (1.00–1.00)	0.899	1.00 (1.00–1.00)	0.381
Obesity, %	0.89 (0.49–1.59)	0.683	0.80 (0.45–1.43)	0.447
eGFR, mL/min/1.73 m^2^	1.01 (1.00–1.02)	0.150	1.01 (0.99–1.02)	0.396

**Table 3:** Competitive risk analysis for the FABP3 and FABP4 concentrations and cardiovascular mortality in subjects with T2D.

	SHR (95% CI)	*p*-Value	SHR (95% CI)	*p*-Value
FABP3, ng/mL	1.28 (1.09–1.50)	0.002	-	-
FABP4, ng/dL	-	-	4.19 (2.21–7.95)	<0.001
Age, years	1.03 (0.99–1.06)	0.101	1.03 (1.00–1.06)	0.059
Sex, women	1.33 (0.57–3.11)	0.516	0.94 (0.38–2.33)	0.886
Ischemic etiology	2.57 (1.26–5.26)	0.010	3.08 (1.42–6.68)	0.004
NYHA III and IV, %	1.98 (0.97–4.04)	0.060	2.42 (1.18–4.96)	0.016
HF duration, years	1.00 (1.00–1.01)	0.867	1.00 (1.00–1.00)	0.937
LVEF	1.00 (0.96–1.03)	0.828	1.00 (0.96–1.03)	0.940
NTproBNP, ng/L	1.00 (1.00–1.00)	0.134	1.00 (1.00–1.00)	0.238
Obesity, %	0.54 (0.22–1.31)	0.171	0.45 (0.19–1.05)	0.065
eGFR, mL/min/1.73 m^2^	1.02 (1.00–1.03)	0.079	1.02 (1.00–1.03)	0.030

**Table 4:** Competitive risks analysis for the FABP3 and FABP4 concentrations and cardiovascular mortality and/or admission for heart failure in subjects with T2D.

	SHR (95% CI)	*p*-Value	SHR (95% CI)	*p*-Value
FABP3, ng/mL	1.14 (0.98–1.32)	0.083	-	-
FABP4, ng/dL	-	-	2.07 (1.11–3.87)	0.022
Age, years	1.04 (1.01–1.07)	0.008	1.04 (1.01–1.07)	0.007
Sex, women	1.20 (0.66–2.16)	0.552	0.96 (0.53–1.76)	0.902
Ischemic etiology	2.21 (1.35–3.60)	0.002	2.31 (1.42–3.74)	0.001
NYHA III and IV, %	1.27 (0.67–2.39)	0.465	1.41 (0.77–2.58)	0.270
HF duration, years	1.00 (1.00–1.00)	0.813	1.00 (1.00–1.00)	0.737
LVEF	0.99 (0.97–1.01)	0.427	0.99 (0.97–1.02)	0.441
NTproBNP, ng/L	1.00 (1.00–1.00)	0.656	1.00 (1.00–1.00)	0.724
Obesity, %	2.14 (1.19–3.89)	0.013	1.88 (1.02–3.46)	0.044
eGFR, mL/min/1.73 m^2^	1.00 (0.99–1.02)	0.702	1.00 (0.99–1.01)	0.625

## Data Availability

Not applicable.

## Supplementary Materials

The following supporting information can be downloaded at: https://www.mdpi.com/article/10.3390/antiox12030645/s1; Figure S1: Heatmaps showing Pearson’s correlations between diabetic lipid parametes (i.e., total-, LDL- and HDL-cholesterol and triglycerides) with FABP3 and FABP4 in T2D (A) and non-T2D (B) patients; Figure S2: Heatmaps showing Pearson’s correlations between urate with FABP3 and FABP4 in T2D (A) and non-T2D (B) patients; Table S1: Cox regression models for the all-cause mortality, FABP3 and FABP4 concentrations in non-T2D; Table S2: Competitive risks models for the cardiovascular mortality, FABP3 and FABP4 concentrations in non-T2D; Table S3: Competitive risks analysis for the FABP3 and FABP4 concentrations and cardiovascular mortality and/or admission for heart failure in subjects with non-T2D; Table S4: Cox regression models for the all-cause mortality, FABP3 and FABP4 concentrations in all the study participants; Table S5: Competitive risks models for the cardiovascular mortality, FABP3 and FABP4 concentrations in all the study participants; Table S6: Competitive risks analysis for the FABP3 and FABP4 concentrations and cardiovascular mortality and/or admission for heart failure in all the study participants.
