# Supplementary material for: Fatty Acid Binding Proteins 3 and 4 Predict Both All-Cause and Cardiovascular Mortality in Subjects with Chronic Heart Failure and Type 2 Diabetes Mellitus
Source: Antioxidants (Basel). 2023 Mar 4;12(3):645. doi: 10.3390/antiox12030645 (PMC10044995; doi:10.3390/antiox12030645)
Supplement: Supplementary file 1 [file antioxidants-12-00645-s001.zip › antioxidants-2252059-supplementary.pdf]

Supplementary Figure S1. Heatmaps showing Pearson's correlations between lipid parametes (i.e. total-, LDL- and HDL-cholesterol and triglycerides) with FABP3 and FABP4 in T2D (A) and non-T2D (B) patients. Pearson's correlations showed that FABP4 inversely correlated with total- (ρ = -0.226, *p*-value < 0.011), LDL- (ρ = -0.186, *p*-value < 0.037) and HDL- cholesterol (ρ = -0.181, *p*-value < 0.042) in non-T2D individuals, but not in T2D patients.

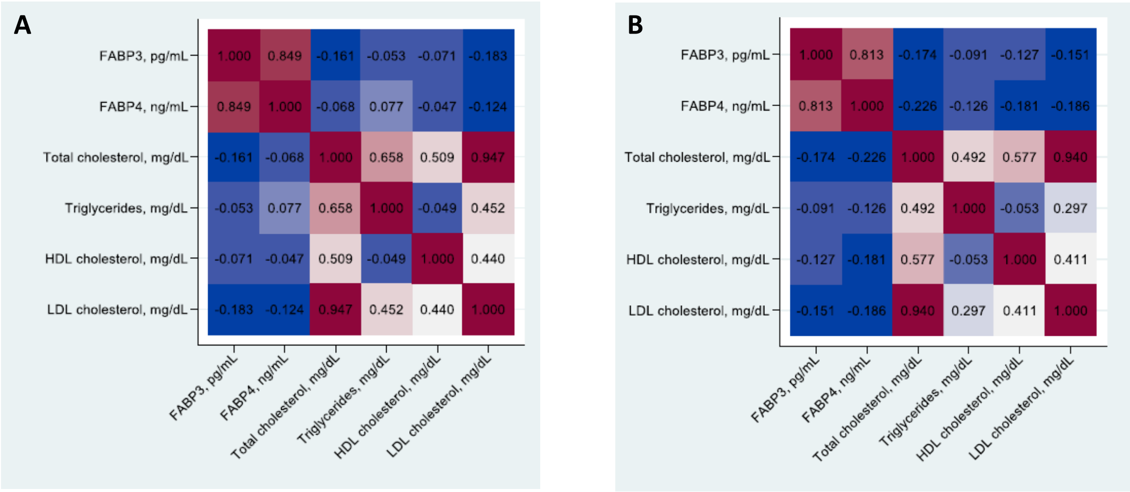

T2D Non-T2D

Supplementary Figure S2. Heatmaps showing Pearson's correlations between urate with FABP3 and FABP4 in T2D (A) and non-T2D (B) patients. Pearson's correlations showed that both FABP3 and FABP4 positively correlated with urate in non-T2D individuals (FABP3: $\rho = 0.221$, *p*-value < 0.013; FABP4: $\rho = 0.195$, *p*-value < 0.029), but not in T2D patients (FABP3: $\rho = 0.022$, *p*-value < 0.820; FABP4: $\rho = -0.038$, *p*-value < 0.692).

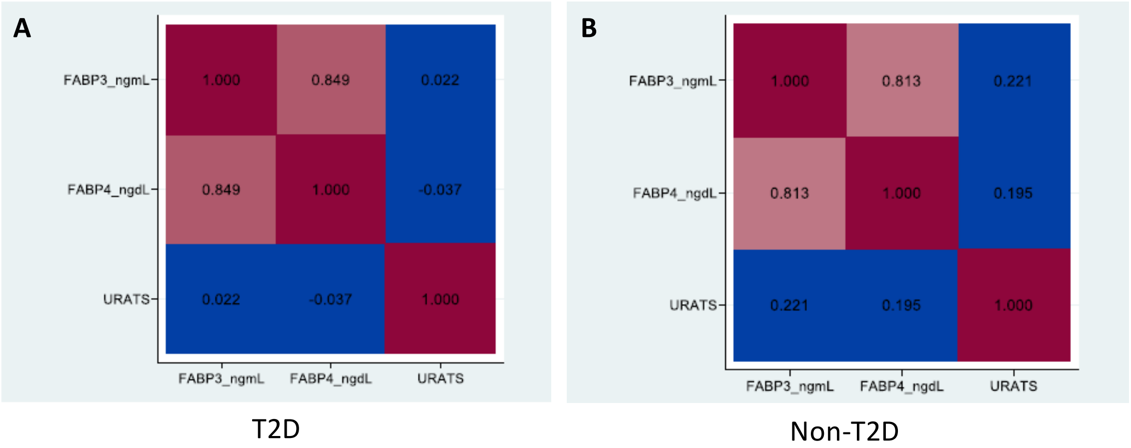

Table S1. Cox regression models for the all-cause mortality, FABP3 and FABP4 concentrations in non-T2D.

	**HR (95% CI)**	***p*-value**	**HR (95% CI)**	***p*-value**
FABP3, ng/mL	1.03 (0.88-1.19)	0.741	-	-
FABP4, ng/dL	-	-	1.20 (0.55-2.61)	0.650
Age, years	1.08 (1.04-1.11)	<0.001	1.08 (1.04-1.11)	<0.001
Sex, women	0.77 (0.38-1.55)	0.461	0.72 (0.34-1.53)	0.390
Ischemic etiology	1.04 (0.55-1.94)	0.911	1.02 (0.55-1.92)	0.940
NYHA III and IV, %	3.47 (1.76-6.84)	<0.001	3.36 (1.66-6.81)	0.001
HF duration, years	1.00 (1.00-1.00)	0.547	1.00 (1.00-1.00)	0.560
LVEF	1.00 (0.98-1.02)	0.903	1.00 (0.98-1.02)	0.924
NTproBNP, ng/L	1.00 (1.00-1.00)	0.271	1.00 (1.00-1.00)	0.167
Obesity, %	0.37 (0.13-1.07)	0.065	0.36 (0.12-1.05)	0.062
eGFR, mL/min/1.73 m^2	0.99 (0.98-1.01)	0.301	0.99 (0.98-1.01)	0.295

Multivariant models have been adjusted for clinically relevant variables. AUC for the FABP3 model = 0.8479. AUC for the FABP4 model = 0.8501. FABP3: fatty acid binding protein 3; FABP4: fatty acid binding protein 4; NYHA: New York heart association; FEECO: ejection fraction on echocardiography; NTproBNP: pro-B-type natriuretic peptide; eGFR estimated glomerular filtration rate (CKD-EPI equation).

Table S2. Competitive risks models for the cardiovascular mortality, FABP3 and FABP4 concentrations in non-T2D

	**SHR (95% CI)**	***p*-value**	**SHR (95% CI)**	***p*-value**
FABP3, ng/mL	1.13 (0.90-1.43)	0.302	-	-
FABP4, ng/dL	-	-	2.22 (0.77-6.37)	0.139
Age, years	1.07 (1.01-1.12)	0.016	1.07 (1.02-1.12)	0.007
Sex, women	0.92 (0.33-2.57)	0.872	0.72 (0.23-2.24)	0.571
Ischemic etiology	1.09 (0.47-2.52)	0.839	1.04 (0.44-2.44)	0.929
NYHA III and IV, %	1.62 (0.57-4.56)	0.363	1.55 (0.55-4.34)	0.402
HF duration, years	1.00 (1.00-1.01)	0.150	1.00 (1.00-1.01)	0.119
LVEF	1.00 (0.97-1.04)	0.959	1.00 (0.97-1.04)	0.779
NTproBNP, ng/L	1.00 (1.00-1.00)	0.822	1.00 (1.00-1.00)	0.920
Obesity, %	0.50 (0.12-2.05)	0.333	0.47 (0.13-1.80)	0.273
eGFR, mL/min/1.73 m^2	1.01 (0.98-1.04)	0.420	1.01 (0.99-1.04)	0.362

Multivariant models have been adjusted for clinically relevant variables. AUC for the FABP3 model = 0.7357. AUC for the FABP4 model = 0.7393. FABP3: fatty acid binding protein 3; FABP4: fatty acid binding protein 4; NYHA: New York heart association; FEECO: ejection fraction on echocardiography; NTproBNP: pro-B-type natriuretic peptide; eGFR estimated glomerular filtration rate (CKD-EPI equation); SHR: Subdistribution Hazard Ratio.

Table S3. Competitive risks analysis for the FABP3 and FABP4 concentrations and cardiovascular mortality and/or admission for heart failure in subjects with non-T2D

	**SHR (95% CI)**	***p*-value**	**SHR (95% CI)**	***p*-value**
FABP3, ng/mL	0.94 (0.78-1.13)	0.518	-	-
FABP4, ng/dL	-	-	0.90 (0.34-2.37)	0.824
Age, years	1.03 (0.99-1.07)	0.109	1.03 (1.00-1.07)	0.081
Sex, women	0.77 (0.37-1.58)	0.474	0.78 (0.38-1.62)	0.506
Ischemic etiology	1.26 (0.70-2.29)	0.438	1.26 (0.68-2.31)	0.460
NYHA III and IV, %	1.52 (0.68-3.43)	0.309	1.47 (0.65-3.35)	0.358
HF duration, years	1.00 (1.00-1.00)	0.147	1.00 (1.00-1.00)	0.158
LVEF	1.02 (1.00-1.04)	0.114	1.02 (0.99-1.04)	0.150
NTproBNP, ng/L	1.00 (1.00-1.00)	0.327	1.00 (1.00-1.00)	0.487
Obesity, %	0.97 (0.43-2.20)	0.945	0.97 (0.43-2.20)	0.941
eGFR, mL/min/1.73 m^2	1.00 (0.98-1.02)	0.720	1.00 (0.98-1.02)	0.935

Multivariant models have been adjusted for clinically relevant variables. AUC for FABP3 model = 0.7036. AUC for FABP4 model = 0.7098. FABP3: fatty acid binding protein 3; FABP4: fatty acid binding protein 4; NYHA: New York heart association; FEECO: ejection fraction on echocardiography; NTproBNP: pro-B-type natriuretic peptide; eGFR estimated glomerular filtration rate (CKD-EPI equation); SHR: Subdistribution Hazard Ratio.

Table S4. Cox regression models for the all-cause mortality, FABP3 and FABP4 concentrations in all the study participants

	**HR (95% CI)**	***p*-value**	**HR (95% CI)**	***p*-value**
FABP3, ng/mL	1.12 (1.02-1.24)	0.018	-	-
FABP4, ng/dL	-	-	1.59 (0.98-2.57)	0.062
Age, years	1.06 (1.04-1.08)	<0.001	1.06 (1.04-1.08)	<0.001
Sex, women	0.66 (0.42-1.03)	0.067	0.58 (0.37-0.91)	0.018
Type 2 Diabetes	1.15 (0.80-1.66)	0.441	1.10 (0.77-1.58)	0.591
Ischemic etiology	1.57 (1.07-2.30)	0.022	1.58 (1.07-2.32)	0.021
NYHA III and IV, %	2.20 (1.46-3.31)	<0.001	2.25 (1.51-3.37)	<0.001
HF duration, years	1.00 (1.00-1.00)	0.263	1.00 (1.00-1.00)	0.273
LVEF	1.01 (0.99-1.03)	0.226	1.01 (0.99-1.03)	0.188
NTproBNP, ng/L	1.00 (1.00-1.00)	0.196	1.00 (1.00-1.00)	0.028
Obesity, %	0.67 (0.42-1.08)	0.100	0.64 (0.40-1.03)	0.067
eGFR, mL/min/1.73 m^2	1.00 (0.99-1.01)	0.872	1.00 (1.00-1.00)	0.752

Multivariant models have been adjusted for clinically relevant variables. AUC for the FABP3 model = 0.8759. AUC for the FABP4 model = 0.8766. FABP3: fatty acid binding protein 3; FABP4: fatty acid binding protein 4; NYHA: New York heart association; FEECO: ejection fraction on echocardiography; NTproBNP: pro-B-type natriuretic peptide; eGFR estimated glomerular filtration rate (CKD-EPI equation).

Table S5. Competitive risks models for the cardiovascular mortality, FABP3 and FABP4 concentrations in all the study participants

	**SHR (95% CI)**	***p*-value**	**SHR (95% CI)**	***p*-value**
FABP3, ng/mL	1.20 (1.06-1.36)	0.004	-	-
FABP4, ng/dL	-	-	2.69 (1.57-4.60)	<0.001
Age, years	1.05 (1.02-1.07)	0.001	1.05 (1.02-1.07)	<0.001
Sex, women	1.13 (0.62-2.06)	0.681	0.84 (0.45-1.56)	0.586
Type 2 Diabetes	1.76 (1.07-2.90)	0.025	1.74 (1.06-2.85)	0.027
Ischemic etiology	1.70 (1.04-2.77)	0.034	1.73 (1.06-2.83)	0.029
NYHA III and IV, %	1.85 (1.07-3.19)	0.027	1.97 (1.17-3.33)	0.011
HF duration, years	1.00 (1.00-1.01)	0.085	1.00 (1.00-1.01)	0.101
LVEF	1.00 (0.97-1.02)	0.816	1.00 (0.98-1.02)	0.923
NTproBNP, ng/L	1.00 (1.00-1.00)	0.243	1.00 (1.00-1.00)	0.418
Obesity, %	0.54 (1.07-2.90)	0.025	0.50 (0.26-0.98)	0.044
eGFR, mL/min/1.73 m^2	1.01 (1.00-1.03)	0.083	1.01 (1.00-1.03)	0.067

Multivariant models have been adjusted for clinically relevant variables. AUC for the FABP3 model = 0.7762. AUC for the FABP4 model = 0.7719. FABP3: fatty acid binding protein 3; FABP4: fatty acid binding protein 4; NYHA: New York heart association; FEECO: ejection fraction on echocardiography; NTproBNP: pro-B-type natriuretic peptide; eGFR estimated glomerular filtration rate (CKD-EPI equation); SHR: Subdistribution Hazard Ratio.

Table S6. Competitive risks analysis for the FABP3 and FABP4 concentrations and cardiovascular mortality and/or admission for heart failure in all the study participants

	SHR (95% CI)	*p*-value	SHR (95% CI)	*p*-value
FABP3, ng/mL	1.04 (0.94-1.16)	0.427	-	-
FABP4, ng/dL	-	-	1.33 (0.82-2.16)	0.253
Age, years	1.04 (1.02-1.06)	<0.001	1.04 (1.02-1.06)	<0.001
Sex, women	1.01 (0.66-1.54)	0.977	0.65 (0.62-1.45)	0.797
Type 2 Diabetes	1.62 (1.11-2.35)	0.012	1.62 (1.12-2.34)	0.010
Ischemic etiology	1.60 (1.12-2.27)	0.009	1.59 (1.12-2.45)	0.009
NYHA III and IV, %	1.42 (0.91-2.22)	0.118	1.43 (0.92-2.21)	0.112
HF duration, years	1.00 (1.00-1.00)	0.256	1.00 (1.00-1.00)	0.271
LVEF	1.00 (0.99-1.02)	0.694	1.00 (0.99-1.02)	0.652
NTproBNP, ng/L	1.00 (1.00-1.00)	0.326	1.00 (1.00-1.00)	0.241
Obesity, %	1.60 (1.04-2.47)	0.033	1.53 (0.99-1.01)	0.557
eGFR, mL/min/1.73 m^2	1.00 (0.99-1.01)	0.625	1.00 (0.99-1.01)	0.523

Multivariant models have been adjusted for clinically relevant variables. AUC for FABP3 model = 0.7615. AUC for FABP4 model = 0.7627. FABP3: fatty acid binding protein 3; FABP4: fatty acid binding protein 4; NYHA: New York heart association; FEECO: ejection fraction on echocardiography; NTproBNP: pro-B-type natriuretic peptide; eGFR estimated glomerular filtration rate (CKD-EPI equation); SHR: Subdistribution Hazard Ratio.
